# Drought, fire, and rainforest endemics: A case study of two threatened frogs impacted by Australia's “Black Summer”

**DOI:** 10.1002/ece3.10069

**Published:** 2023-05-19

**Authors:** Geoffrey W. Heard, Liam J. Bolitho, David Newell, Harry B. Hines, Patrick Norman, Rosalie J. Willacy, Ben C. Scheele

**Affiliations:** ^1^ Fenner School of Environment and Society Australian National University Canberra Australian Capital Territory Australia; ^2^ Terrestrial Ecosystem Research Network University of Queensland Brisbane Queensland Australia; ^3^ Centre for Biodiversity and Conservation Science University of Queensland Brisbane Queensland Australia; ^4^ Faculty of Science and Engineering Southern Cross University Lismore New South Wales Australia; ^5^ Department of Environment and Science Queensland Parks and Wildlife Service and Partnerships Bellbowrie Queensland Australia; ^6^ Queensland Museum South Brisbane Queensland Australia; ^7^ Climate Action Beacon Griffith University Gold Coast Queensland Australia

**Keywords:** amphibian, climate change, drought, Gondwanan, megafire, Pyrocene

## Abstract

Deepening droughts and unprecedented wildfires are at the leading edge of climate change. Such events pose an emerging threat to species maladapted to these perturbations, with the potential for steeper declines than may be inferred from the gradual erosion of their climatic niche. This study focused on two species of amphibians—*Philoria kundagungan* and *Philoria richmondensis* (Limnodynastidae)—from the Gondwanan rainforests of eastern Australia that were extensively affected by the “Black Summer” megafires of 2019/2020 and the severe drought associated with them. We sought to assess the impact of these perturbations by quantifying the extent of habitat affected by fire, assessing patterns of occurrence and abundance of calling males post‐fire, and comparing post‐fire occurrence and abundance with that observed pre‐fire. Some 30% of potentially suitable habitat for *P. kundagungan* was fire affected, and 12% for *P. richmondensis*. Field surveys revealed persistence in some burnt rainforest; however, both species were detected at a higher proportion of unburnt sites. There was a clear negative effect of fire on the probability of site occupancy, abundance and the probability of persistence for *P. kundagungan*. For *P. richmondensis*, effects of fire were less evident due to the limited penetration of fire into core habitat; however, occupancy rates and abundance of calling males were depressed during the severe drought that prevailed just prior to the fires, with the reappearance of calling males linked to the degree of rehydration of breeding habitat post‐fire. Our results highlight the possibility that severe negative impacts of climate change for montane rainforest endemics may be felt much sooner than commonly anticipated under a scenario of gradual (decadal‐scale) changes in mean climatic conditions. Instead, the increased rate of severe stochastic events places these narrow range species at a heightened risk of extinction in the near‐term.

## Graphical abstract

This paper documents the effects of the 2019/2020 “Black Summer” fires and the associated unprecedented drought on two species of threatened rainforest frogs from eastern Australia. This study demonstrates important impacts of these climate‐forced events on these two species, including significant fire penetration into mesic rainforest habitat and drought‐induced population contractions. The work demonstrates the susceptibility of montane endemics to drought and fire under climate change, and the potential for step‐change reductions in occurrence that may not be predicted from erosion of the climatic niche alone.

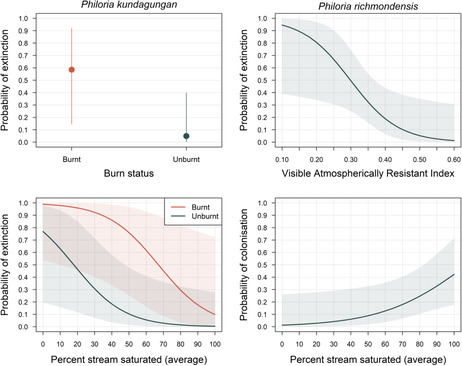

## 1 INTRODUCTION

It is now clear that severe droughts and unprecedented wildfires are at the leading edge of climate change, and will threaten some species long before forecast erosion of their climatic niche (Kelly et al., 2020). The burgeoning risk of wildfire with the accumulation of carbon dioxide in the atmosphere has been predicted for some time (Scholze et al., 2006). Longer and more extreme fire seasons have been predicted for already fire‐prone regions, resulting from increasing temperatures, more erratic rainfall, deeper droughts, and heatwaves (Hennessy et al., 2005; Moriondo et al., 2006; Pitman et al., 2007). However, intense droughts and concomitant fire events are increasingly impacting regions and ecosystems in which they were historically extremely rare or unknown, including tropical and sub‐tropical rainforests in Australia, south‐east Asia, and South America (Barlow et al., 2020; Chisholm et al., 2016; Collins et al., 2021).

Montane species are particularly threatened by climate change. The typically small range and narrow climatic niche of these species leaves them inherently susceptible (Ohlemüller et al., 2008), as does their lower thermal tolerances and adaptation to moist environments (McCain & Colwell, 2011). Studies on the impacts of climate change on montane species have subsequently focused on these mechanisms, often aided by forward‐projection of environmental niche models. In Australia, for example, significant effort has been expended over the last two decades building environmental niche models to estimate the scale and pace of range contractions for montane species under climate change, with focus on gradual changes to temperature and rainfall profiles (Bateman et al., 2012; Bond et al., 2011; Costion et al., 2015; Das et al., 2019; Fordham et al., 2016; Kearney et al., 2010; Sopniewski et al., 2022; Williams et al., 2003). While an important and valid approach, these studies usually exclude alteration of drought and fire regimes under climate change, at least explicitly. Recent increases in the frequency, extent, and severity of wildfire across Australia in the last two decades (Abram et al., 2021)—including megafires that have penetrated ecosystems never previously known to burn (Hines et al., 2020; Holz et al., 2015)—suggest that sharp changes in fire regimes driven by severe drought may be crucial to such projections.

The “Black Summer” megafires across eastern Australia during 2019/2020 highlighted the risk that unprecedented drought and fire poses to Australia's biodiversity under climate change (Legge et al., 2022; Rumpff et al., 2023; Ward et al., 2020). These fires burnt ~10 M hectares of woodland, forest, and rainforest during Australia's hottest and driest year on record (Collins et al., 2021; Filkov et al., 2020). The radiative power of these fires, the area burnt at high severity, and the area of rainforest burnt, were all without precedence in the historical record (Abram et al., 2021; Collins et al., 2021; Rumpff et al., 2023). In northern New South Wales and southeast Queensland, some 53% of the Gondwana Rainforests of Australia World Heritage Area was affected by fire during the 2019/2020 event (DAWE, 2020a). While the majority of these impacts occurred in fire‐adapted eucalypt forest, a considerable area of fire‐sensitive subtropical, warm‐temperate, and cool‐temperature rainforest was also burnt (DAWE, 2020a).

The Gondwanan rainforests of northern New South Wales and southern Queensland support several species of endemic, range‐restricted amphibians that contribute to the outstanding universal values of the World Heritage area. Notable among these are six of seven species in the genus *Philoria* (Limnodynastidae), being *P. knowlesi*, *P. kundagungan*, *P. loveridgei*, *P. pughi*, *P. richmondensis*, and *P. sphagnicolus* (Knowles et al., 2004; Mahony et al., 2022). These species are all allopatric, occurring as scattered mountain‐top endemics restricted to headwater drainage lines, seepages, and small bogs in rainforest and adjoining mesic vegetation (Knowles et al., 2004, Mahony et al., 2022). Males construct and call from nests in saturated soil. Tadpoles complete their development within the nest and their survival is dependent on constant moisture (Anstis, 2013). Microhabitat use outside the breeding season is almost totally unknown; however, radio‐tracking studies on the southern member of the genus from Victoria, *P. frosti*, indicate that both males and females likely migrate small distances (<85 m) away from seepages post‐breeding to occupy moist microhabitats slightly upslope (Hollis, 2003).

The reliance of *Philoria* on moist or saturated microhabitats within rainforest and adjoining mesic forest, suggests that they may be particularly sensitive to increased drought and novel fire under climate change (Bolitho & Newell, 2022; Mahony et al., 2023; Newell, 2018). This study sought to assess the impact of the “Black Summer” fires and the severe drought associated with them on two species from the Gondwana Rainforests of Australia World Heritage Area—*P. kundagungan* and *P. richmondensis*. Both are listed as Endangered by the International Union for the Conservation of Nature (IUCN, 2021) and the Australian Government (DCCEEW, 2022).

We had four aims for each species: (1) estimate the portion of potential habitat affected by the 2019/2020 fires; (2) resurvey all previous survey sites post‐fire, adding new sites in fire‐impacted catchments; (3) examine the effects of fire and drought on site occupancy probability and abundance of calling males, and; (4) assess changes in site occupancy and calling male abundance from surveys completed pre‐fires, and determine the roles of drought and fire on these dynamics.

## 2 MATERIALS AND METHODS

### 2.1 Study area and survey sites

**FIGURE 1:**
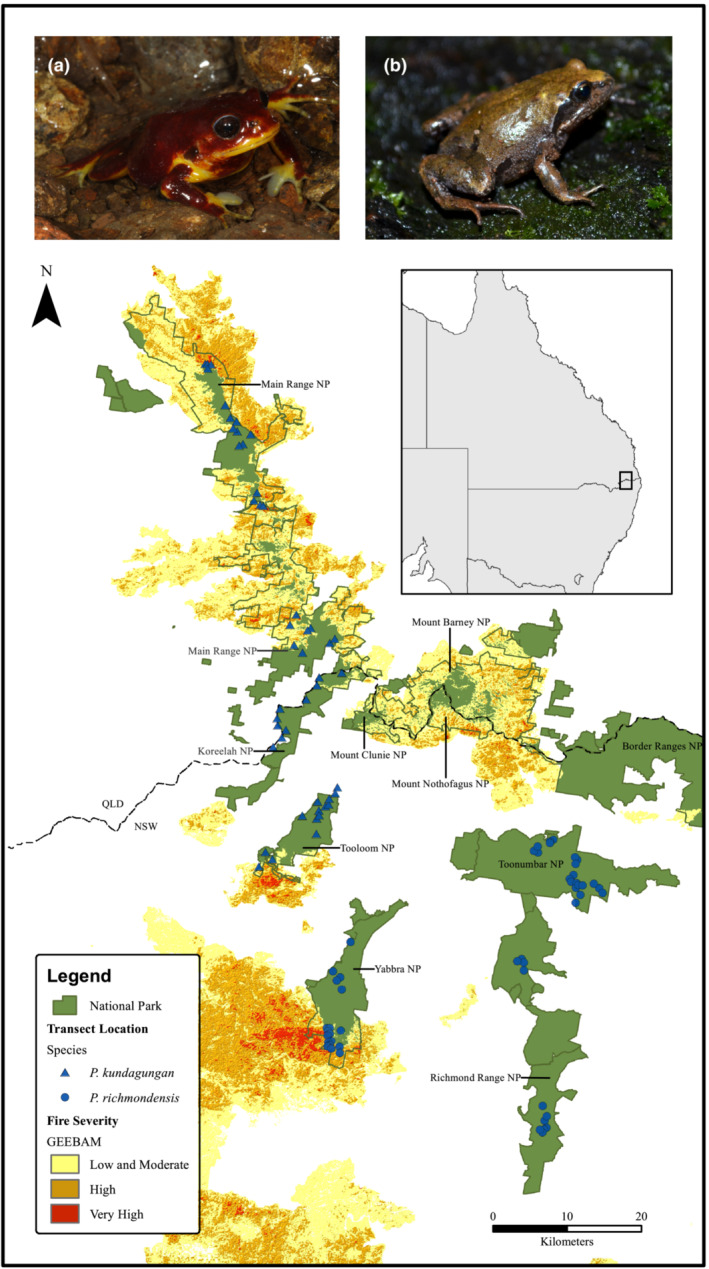
Map of the study area, showing locations of transects surveyed for *Philoria kundagungan* (a) and *Philoria richmondensis* (b), and their positions relative to the extent and severity of fire during the “Black Summer” wildfires of 2019/2020.

The study area extended across the uplands of north‐eastern New South Wales and south‐eastern Queensland (Figure 1). In the east, surveys were completed for *P. richmondensis* in Yabbra, Richmond Range and Toonumbar National Parks. In the west and north‐west of the study area, surveys were completed for *P. kundagungan* in Tooloom and Koreelah National Parks in New South Wales, and along the length of Main Range National Park in Queensland.

Sites were defined as 100 m transects along headwater streams (1st or 2nd order) beginning from the first expression of surface water. An original set of sites for *P. richmondensis* (*n* = 37) was established in 2012 with the aid of a species distribution model (Willacy, 2014). Sites were randomly located in 12 headwater stream catchments that displayed a predicted habitat suitability of 40% or greater, under the criterion that they were at least 200 m apart and within 500 m of a road or trail. An original set of sites for *P. kundagungan* (*n* = 35) was established by Bolitho et al. (2021) in 2016. Sites were randomly selected across the range of this species under three constraints: (1) they were within 500 m of rainforest; (2) they were >500 m from another site, and; (3) they were within 2 km of a road or operational fire trail.

To increase coverage of habitat burnt during the 2019/2020 fires, we added a further 13 sites for *P. kundagungan* at the northern and southern extremities of Main Range National Park, and a further 13 sites for *P. richmondensis* at the southern end of Yabbra National Park (the only area within the species' range that was affected by these fires). In total, 48 sites were surveyed for *P. kundagungan* (14 burnt) and 50 sites surveyed for *P. richmondensis* (13 burnt).

### 2.2 Surveys

Surveys were completed for calling males between September 2020 and February 2021; the breeding season immediately following the “Black Summer” fires. Both species call primarily in the Austral spring, with a peak of calling in September–October and gradual reduction in calling as temperatures increase in summer (Bolitho et al., 2021; Willacy et al., 2015). Up to five repeat surveys were completed at each site (median = 3 surveys, total surveys = 285), with the vast majority completed by a single experienced field‐worker. Surveys were undertaken during daylight hours, as calling activity is minimal at night (Bolitho et al., 2021; Willacy et al., 2015). Surveys entailed quietly walking the transect for a minimum of 15 min, while eliciting calls by broadcasting male advertisement calls with a hand‐held speaker for ~1 min at each 10 m increment. The locations of individual calling males are easily distinguished for both *P. kundagungan* and *P. richmondensis*, due to the low density of calling males along transects (≤0.025 males per square metre) and the discrete nature of nesting sites. The total number of males calling, the date of survey, start and end times, and five variables that may influence calling rate were recorded during each survey: ambient air temperature, water temperature, relative humidity, cloud cover, and precipitation level. Weather variables were measured with the aid of a Kestrel weather meter and probe thermometer (various models in each case).

Surveys in New South Wales were completed under scientific license SL102444 and Southern Cross University animal ethics permit 20/036. Surveys in Queensland were completed under authority from the Queensland Department of Agriculture and Fisheries Community Animal Ethics Committee, permit SA 2019/08/700.

### 2.3 Field assessments of fire severity and drought stress

**FIGURE 2:**
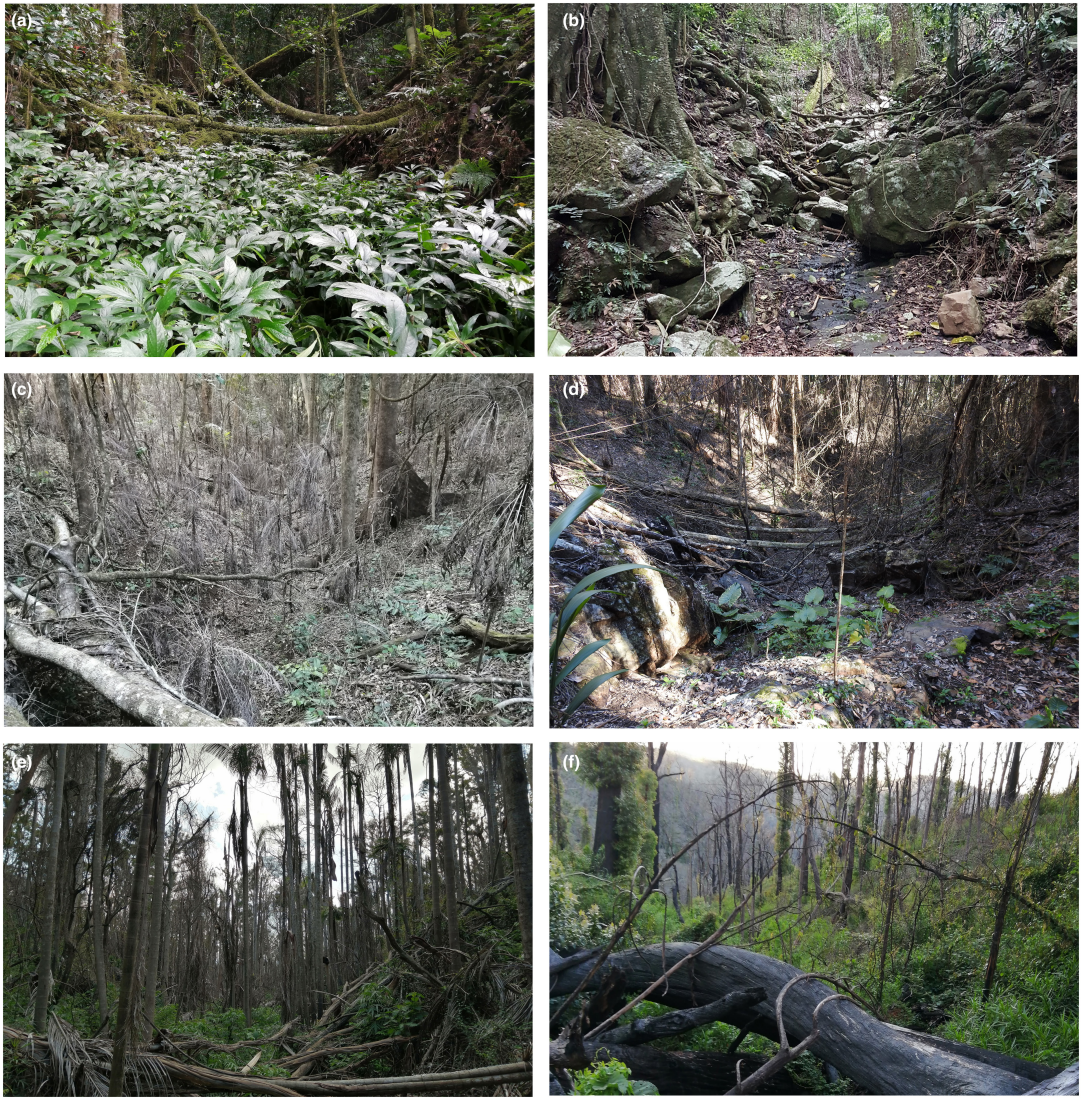
Sites at which surveys for *Philoria kundagungan* and *Philoria richmondensis* were completed during the 2020/2021 season, showing examples of unburnt habitat, habitat burnt at different severity levels, and drought affected and unaffected habitat at the time of survey. (a) Unburnt and not drought affected; (b) Unburnt but drought affected; (c) Burnt at low–moderate severity and drought affected; (d) Burnt at moderate severity and drought affected; (e) Burnt at moderate–high severity, but no longer drought affected; (f) Burnt at high severity and drought affected.

Fire impacts at each site were scored using a suite of measures. The percentage of the stream bed burnt and the percentage of the bank burnt were estimated while pacing the entire transect after the first survey. For each 10 m section, the percentage of the stream bed with evidence of fire was estimated, from which a total percentage of the stream bed burnt could be derived. Likewise, the percentage of the bank burnt on both sides of the stream to 10 and 20 m perpendicular to the stream bed was estimated for each 10 m section of the transect. This allowed the total percentage of the bank burnt on each side of the stream to be estimated for each distance class. Fire severity was also scored on an ordinal scale in each of these zones on the stream bank, as either: (1) “Unburnt”; (2) “Low” (canopy and subcanopy unscorched, shrubs may be scorched, fire‐sensitive low shrubs may be killed); (3) “Moderate” (partial canopy scorch, subcanopy partially or completely scorched, and/or fire‐sensitive tall shrub or small tree layer mostly killed); (4) “High” (full canopy scorch to partial canopy consumption, subcanopy fully scorched, or consumed), or; (5) “Extreme” (full canopy, subcanopy, and understorey consumption). In situations where different levels of fire severity were present in a stream bank zone, the severity category that covered the largest proportion of the burnt area of the zone was assigned. Sites burnt at varying levels of severity are depicted in Figure 2.

A suite of additional environmental variables was measured at each site, although only a subset was considered here that relate to drought stress. The percentage of the stream bed saturated was estimated during each survey, as were the percentage of the stream with surface water and the percentage of the stream supporting clumps of rainforest spinach (*Elatostema* spp.); an important microhabitat for northern *Philoria* species (Bolitho et al., 2021; Willacy, 2014) that is highly sensitive to desiccation. These estimates were derived using the same approach as that applied for estimating to percentage of the stream bed burnt (as above).

### 2.4 Remote‐sensed variables

To complement field‐based measures of fire severity at each site, the Australian Google Earth Engine Burnt Area Map (GEEBAM; DAWE, 2020b) was acquired as a 30 m resolution raster layer and the average burn severity (scale = 0–24) estimated for each site at two spatial scales: (1) within a 20 m buffer surrounding each stream transect, and; (2) within a 100 m buffer of the transect midpoint. Both were strongly correlated with burn severity measured during site assessments (*r* > .9 when tested against the scored burn severity of the banks to 20 m).

Two remote‐sensed measures of drought‐severity were also derived, both of which are spectral indices known to be strongly correlated with water availability for vegetation (Caccamo et al., 2012). The visible atmospherically resistant index (VARI; Gitelson et al., 2002) is sensitive to vegetation greenness, whereas the normalized difference infrared index centered on 1650 nm (NDIIb6) is sensitive to vegetation water content (Caccamo et al., 2012). Layers of both indices were calculated using Landsat 8 imagery at a 30 m resolution for August 2019, immediately prior to the 2019/2020 fires. The indices were standardized against 5 years of data (August 2014–July 2019) through spectral index normalization to calculate habitat drought stress during the period just before the fires. Both drought severity indices were measured for each site at the two spatial scales used for the remote‐sensed indices of fire severity.

Lastly, elevation (m asl) of the transect mid‐point was estimated from a 5 m resolution digital elevation model from Geosciences Australia. Site elevation is a strong predictor of habitat suitability for both *P. kundagungan* (Bolitho et al., 2021) and *P. richmondensis* (Willacy, 2014) and therefore was an important covariate for modeling the effects of drought and fire on these species.

### 2.5 Data analysis

To assess the proportion of suitable habitat burnt during the fires, we first identified streams potentially occupied by each species using the approach of Bolitho et al. (2021). Potential habitat for each species was defined as sections of these streams rasterized to 90 m × 90 m pixels to capture areas of adjoining forest likely to be utilized by *Philoria* (measured at up to 85 m from breeding sites for *P. frosti*; Hollis, 2003). The extent of fire within potential habitat was assessed using the GEEBAM layer, with the aid of the *sf* package for *R* version 4.0.2 (R Core Team, 2021).

From the wider pool of site‐level variables, we selected 10 for inclusion in the analysis of survey data, guided by previous studies (Bolitho et al., 2021; Willacy, 2014) and visual assessment of relationships between these variables and the frog survey data. The 10 variables were elevation, whether sites were burnt or unburnt (based on visual evidence of fire in or immediately adjacent to the stream bed), percentage of the stream bed burnt, average bank burn severity score (unburnt = 0, low = 1, moderate = 2, high = 3, extreme = 4) across both stream banks, average GEEBAM burn severity within a 100 m buffer of the transect center, percentage of stream with rainforest spinach, average and minimum percentage of the stream saturated, and average drought severity (NDIIb6 and VARI) within a 20 m buffer of the transect center.

Single‐season occupancy models (MacKenzie et al., 2006) were fitted to the survey data for each species. Fifty candidate models were considered, each with alternate additive effects of the 10 chosen variables on the probability of site occupancy (*ψ*) by calling males. Elevation was included in all models a priori and up to four additive combinations of the remaining variables included (only those variables with pairwise correlation coefficients of <.5 were included in the same model). The per‐survey detection probability (*p*) was set as a function of air temperature at the time of survey in all cases after Bolitho et al. (2021). Covariate effects were modeled with a linear equation and logistic link for both *ψ* and *p*. Candidate models were fitted to the data for each species with the aid of the *unmarked* package for *R* (Chandler et al., 2021).

The same candidate model set was fitted to counts of calling males during each survey using generalized linear mixed models (GLMMs). Counts were assumed to follow a Poisson distribution, with the mean count for each species (*λ*) modeled as a function of site‐ and survey‐level covariates using a log‐linear equation. As for occupancy models, only air temperature was included as a predictor of detection rate at the time of survey. Candidate models were fitted sequentially for both species in *R* using the *lme4* package (Bates et al., 2021), with all models including a site‐based random effect to account for repeated counts.

Surveys conducted prior to the 2019/2020 fires were used to assess changes in site occupancy and abundance of calling males, and to assess the role of fire and drought in these dynamics. For *P. kundagungan*, the original surveys of Bolitho et al. (2021) completed at 35 of the focal sites during the 2016/2017 breeding season provided the basis for these comparisons. For *P. richmondensis*, two earlier seasons of survey data were available for 37 of the focal sites; the original surveys of Willacy (2014) completed in the 2012/2013 season and repeat surveys at these sites in 2019 just prior to the Black Summer fires (L. Bolitho and D. Newell, unpublished). These surveys followed the same approach as that undertaken during the 2020/2021 field season (repeat surveys conducted from September to January), allowing for direct comparisons.

Changes in site occupancy were assessed using dynamic occupancy models (MacKenzie et al., 2006) fitted to the data with the aid of the *unmarked* package in *R*. Within species, models varied only in their structure for the probability of extinction between survey years (*ε*), but differed slightly between species due to differences in the available data. For *P. kundagungan*, we fitted models with effects of elevation, fire and drought on the probability of extinction (2016/2017 → 2020/2021), with variable inclusion guided by model selection results for the single‐season analysis described above. Fire was represented by the binary indicator variable of burnt or not in 2019/2020 and the remote‐sensed GEEBAM fire severity scores. Drought was represented by percentage of the stream bed saturated in 2020/2021 and the two remote‐sensed measures of drought‐severity (NDIIb6 and VARI). For *P. richmondensis*, only elevation and the two remote‐sensed drought variables were included as covariates of the probability of extinction, as none of the sites surveyed in all three seasons were burnt in 2019/2020 and stream saturation extent was only measured in 2020/2021. In all cases, the probability of site occupancy in the first season (*ψ*) was set as a function of elevation alone (this being the top‐model in the studies of Bolitho et al., 2021 and Willacy, 2014). The probability of colonization (*γ*) was held constant for *P. kundagungan* given that there was only a single observed colonization event (i.e., not detected at a site in 2016/2017 and then detected in 2020/2021). For *P. richmondensis*, the probability of colonization was modeled as a function of stream saturation extent measured in 2020/2021, given that almost all observed colonizations occurred from 2019/2020 to 2020/2021, and moisture availability was considered a priori to be an important proximate driver of this probability. As for the single‐season analysis, the per‐survey detection probability was modeled as a function of air temperature at the time of survey and only variables with pairwise correlation coefficients <.5 were included in the same model.

All data and code necessary to reproduce the analysis are provided in Heard et al. (2022).

## 3 RESULTS

### 3.1 Extent of habitat burnt

The area of potential habitat for *P. kundagungan* was estimated at 69.5 sq. km, of which 21.1 sq. km (30%) was burnt during the 2019/2020 fires. Of the burnt area, 71% was burnt at low–moderate severity, 27% was burnt at high severity, and 2% was burnt at very high severity (based on GEEBAM classifications). For *P. richmondensis*, 11.9 sq. km of the 96.8 sq. km of potential habitat was burnt (12%), with 45% of the burnt area experiencing low–moderate severity fire, 42% experiencing high severity fire, and 13% experiencing very high severity fire.

### 3.2 Site occupancy post‐fire

Fire severity among the 14 burnt sites for *P. kundagungan* was generally low to moderate; however, five sites experienced >90% of the stream bed or banks burnt, with two of these experiencing high severity fire. Surveys detected *P. kundagungan* at 25 of 48 sites (52%), with the species detected at only 2 of the 14 burnt sites (14%) compared to 23 of the 34 unburnt sites (68%). For *P. richmondensis*, 12 of the 13 burnt sites experienced moderate to high severity fire, with an average of 98% of the stream bed burnt and 99.7% of the banks burnt. *Philoria richmondensis* was detected at 23 of 50 sites (46%), with detection at 3 of the 13 burnt sites (23%) compared with detection at 20 of 37 unburnt sites (54%).

**FIGURE 3:**
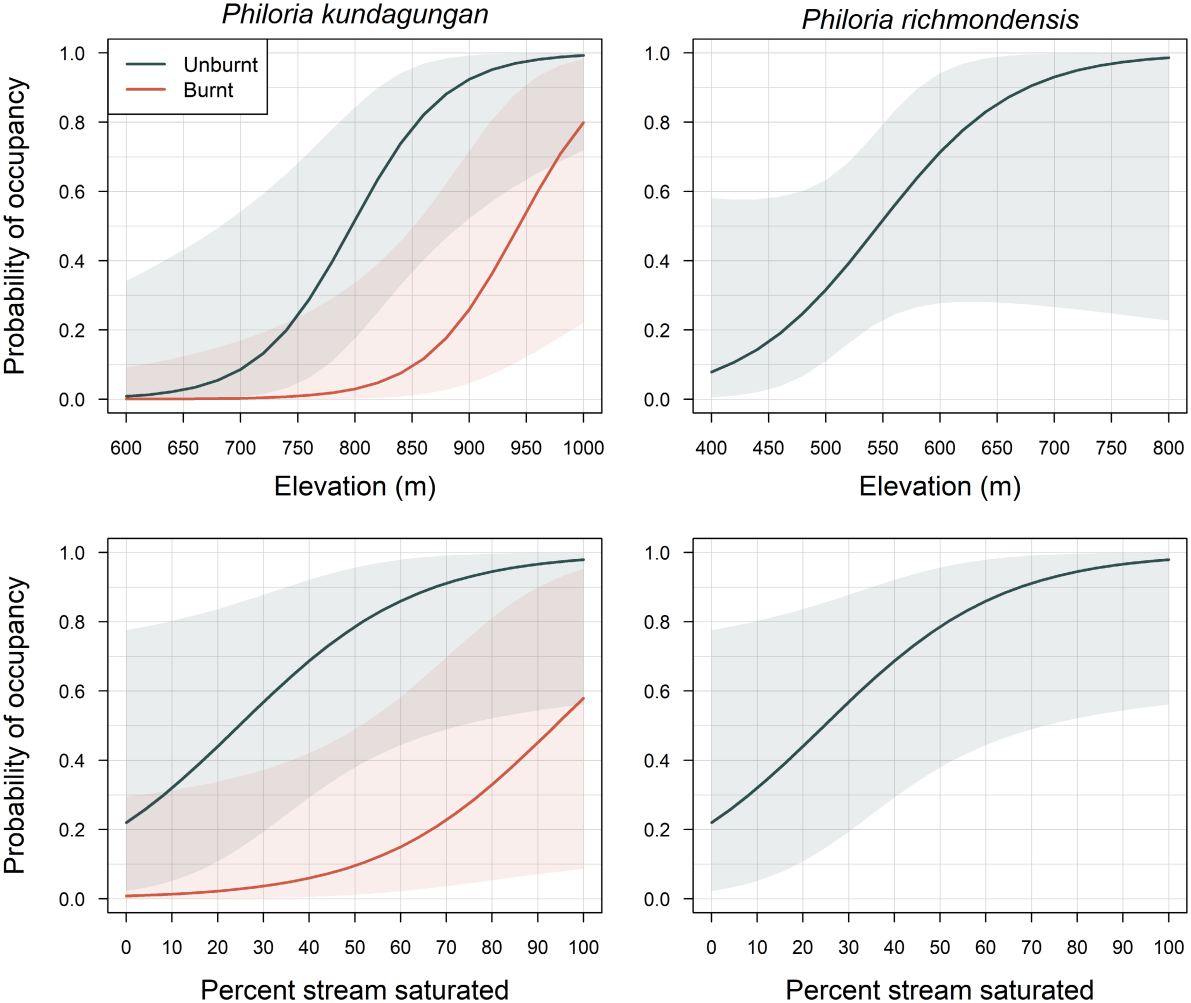
Relationships between the probability of site occupancy and both elevation and percentage of stream saturated for *Philoria kundagungan* and *Philoria richmondensis* post‐fire. Relationships are depicted for burnt and unburnt sites for *P. kundagungan*, with burn status being a key predictor of occupancy probability for this species. Relationships for elevation and percentage of stream saturated are shown with the other covariate held at its mean. Percent stream saturated is the average value across repeat surveys at a site. Shaded areas are 95% confidence intervals.

Model selection statistics indicate an important effect of fire on site occupancy probability for *P. kundagungan*, with the top five models for this species including a negative effect of fire on this probability (Tables 1 and 2, Figure 3). Two of the three top models also included a positive effect of percent stream saturation on site occupancy probability for *P. kundagungan*, with the second top model including a weak negative effect of remote‐sensed drought severity (variable NDIIb6) prior to the 2019/2020 fires (Tables 1 and 2, Figure 3). Clear effects of fire on site occupancy probability were not apparent for *P. richmondensis*. Site elevation and percent stream saturation were the most influential variables for site occupancy probability, with strong positive effects in both cases (Tables 1 and 2, Figure 3). Effects of fire appeared in the third, fourth, and fifth top models; however, the effects were weak and these models had considerably less support than the top two models excluding fire effects (ΔAIC >2; Table 1). The survey‐level probability of detection declined steeply with increasing air temperature for both *P. kundagungan* and *P. richmondensis* (Table 2).

**TABLE 1:** The top five site occupancy models for *Philoria kundagungan* and *Philoria richmondensis* post‐fire. Percent stream saturated is the average value across repeat surveys at a site, except where indicated otherwise.

Model	Log likelihood	AIC	ΔAIC	Model weight
*P. kundagungan*				
Elevation, burn status, percent stream saturated	−50.71	115.47	0.00	0.19
Elevation, burn status, drought severity (NDIIb6)	−51.08	116.22	0.74	0.13
Elevation, GEEBAM fire severity, percent stream saturated	−51.34	116.72	1.25	0.10
Elevation, burn status	−52.92	117.27	1.79	0.08
Elevation, burn status, cover of rainforest spinach, percent stream saturated	−50.51	117.82	2.35	0.06
*P. richmondensis*				
Elevation, percent stream saturated	−52.07	115.51	0.00	0.25
Elevation, percent stream saturated (minimum)	−52.88	117.13	1.62	0.11
Elevation, GEEBAM fire severity, percent stream saturated	−51.78	117.52	2.01	0.09
Elevation, bank burn severity, percent stream saturated	−51.95	117.85	2.34	0.08
Elevation, burn status, percent stream saturated	−52.05	118.06	2.56	0.07

*Note*: Akaike's information criterion (AIC) is provided for each model, as is distance from the top model (ΔAIC). Elevation was included in all models a priori, with an effect of air temperature on detection probability also common to all models.

**TABLE 2:** Coefficient estimates from the top site occupancy models for *Philoria kundagungan* and *Philoria richmondensis* post‐fire. Percent stream saturated is the average value across repeat surveys at a site.

Variable	Covariate of	*P. kundagungan*		*P. richmondensis*	
		Mean	95% CI	Mean	95% CI
Elevation	Occupancy probability	5.16	1.20, 9.13	2.96	−0.5, 6.43
Burn status	Occupancy probability	−3.55	−6.71, −0.38	‐	‐
Percent stream saturated	Occupancy probability	3.12	0.02, 6.21	6.00	1.50, 8.70
Air temperature	Detection probability	−3.03	−4.69, −1.38	−2.87	−4.25, −1.48

*Note*: Mean estimates are provided along with the 95% confidence interval (95% CI).

### 3.3 Abundance of calling males post‐fire

Counts of calling males were generally low at sites occupied by *P. kundagungan* (62% of counts at occupied sites being ≤5 individuals). However, 10 or more individuals were detected on 11 occasions, with a maximum count of 22 in a single site survey. Maximum counts at the two burnt sites at which *P. kundagungan* was detected were 4 and 10, while at unburnt sites counts averaged 7 individuals, with a range of 1–22. At the three burnt sites at which *P. richmondensis* was detected, maximum counts of calling males were 1, 3, and 14, with the latter being at a site that was burnt with very low severity. Counts of *P. richmondensis* at unburnt sites averaged 5, with a range of 1–14.

Rankings of the candidate models when fitted to the count data for each species were very similar to those for the occupancy models (Table 3). Counts of calling males increased with elevation and percent stream saturated for both *P. kundagungan* and *P. richmondensis*, and declined with increasing air temperature at the time of survey (Table 4). Burn status (burnt or unburnt) again featured in the top model for *P. kundagungan*; however, the effect was weaker than that for the probability of site occupancy, with the model including it being only slightly superior to the second ranked model that excluded it (Table 3) and the 95% confidence interval for the effect overlapping zero (Table 4). While burn status also featured in the top model for *P. richmondensis* (Table 3), the effect was weakly positive (Table 4) due to an outlying count of 14 calling males at one very lightly burnt site.

**TABLE 3:** The top five models of counts of calling males for *Philoria kundagungan* and *Philoria richmondensis* post‐fire. Percent stream saturated is the average value across repeat surveys at a site.

Model	Log likelihood	AIC	ΔAIC	Model weight
*P. kundagungan*				
Elevation, burn status, percent stream saturated	−209.70	432.03	0.00	0.24
Elevation, percent stream saturated	−211.02	432.50	0.47	0.19
Elevation, cover of rainforest spinach, percent stream saturated	−210.47	433.57	1.55	0.11
Elevation, bank burn severity, percent stream saturated	−210.66	433.95	1.93	0.09
Elevation, burn status, cover of rainforest spinach, percent stream saturated	−210.66	433.95	1.93	0.09
*P. richmondensis*				
Elevation, burn status, percent stream saturated	−156.21	325.02	0.00	0.21
Elevation, percent stream saturated	−157.42	325.28	0.26	0.19
Elevation, GEEBAM fire severity, percent stream saturated	−156.74	326.08	1.06	0.13
Elevation, burn status, cover of rainforest spinach, percent stream saturated	−155.92	326.64	1.63	0.10
Elevation, bank burn severity, percent stream saturated	−157.36	327.32	2.30	0.07

*Note*: Akaike's information criterion (AIC) is provided for each model, as is distance from the top model (ΔAIC). Air temperature (not shown) was included in all models given its effect on calling rate.

**TABLE 4:** Coefficient estimates for the top models of counts of calling males for *Philoria kundagungan* and *Philoria richmondensis* post‐fire. Percent stream saturated is the average value across repeat surveys at a site.

Variable	*P. kundagungan*		*P. richmondensis*	
	Mean	95% CI	Mean	95% CI
Elevation	1.84	0.60, 3.42	1.16	0.49, 1.92
Burn status	−1.00	−2.45, 0.20	0.88	−0.26, 1.87
Percent stream saturated	2.35	1.26, 3.56	3.19	2.08, 4.50
Air temperature	−1.67	−2.08, −1.27	−2.01	−2.59, −1.46

*Note*: Mean estimates are provided along with the 95% confidence interval (95% CI).

### 3.4 Change in site occupancy and abundance from pre‐fire

**FIGURE 4:**
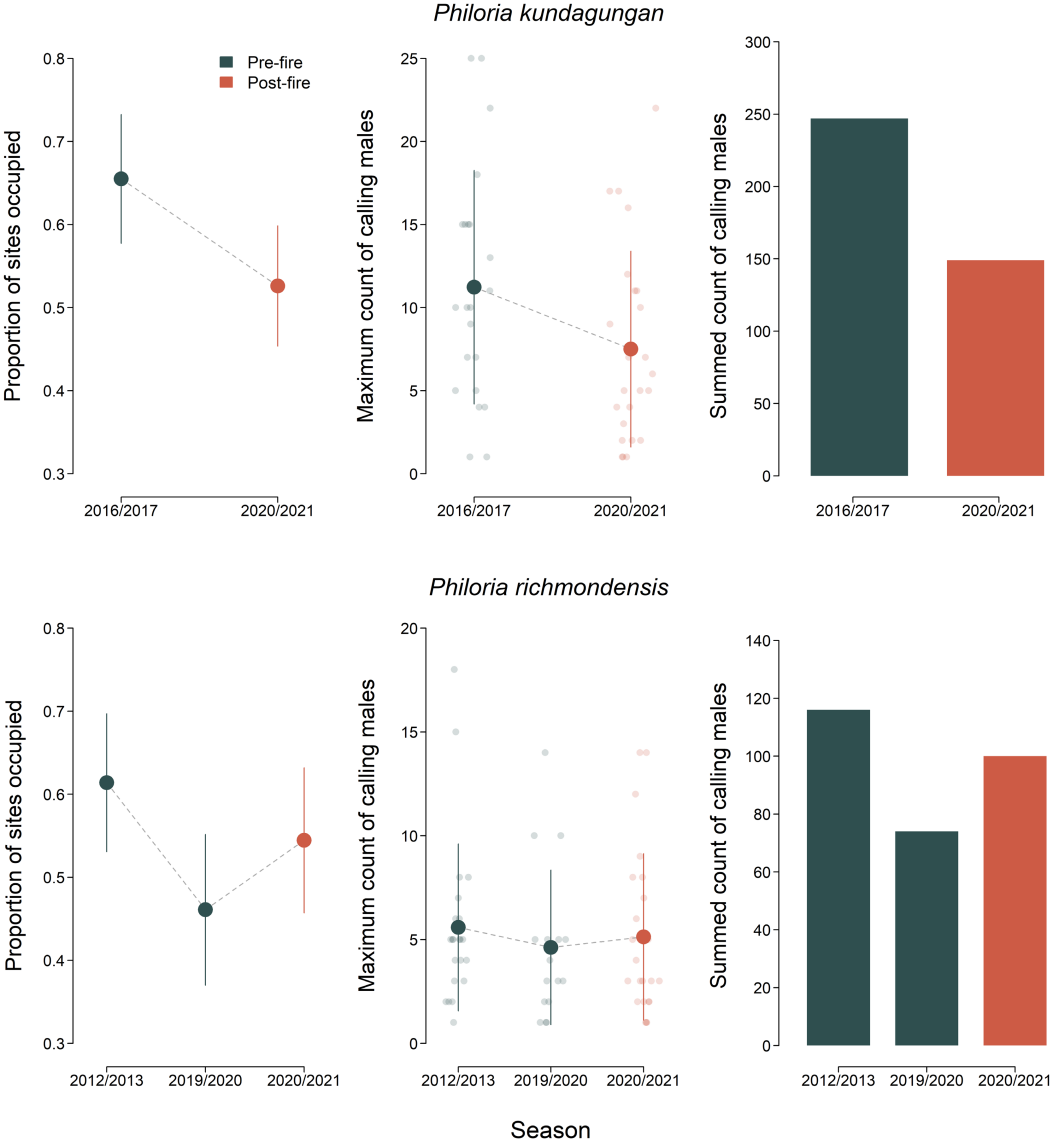
Comparison of the proportion of sites occupied and counts of *Philoria kundagungan* and *Philoria richmondensis* during the 2020/2021 season with those available pre‐fire. Data are from the subset of sites surveyed in all seasons for each species (*n* = 35 for *P. kundagungan* and *n* = 37 for *P. richmondensis*). The left‐hand plots show estimates of the proportion of sites occupied in each season, with vertical lines being ±1 standard error. The middle plots show maximum counts of calling males at occupied sites in each season. Raw data are shown as jittered points, with large dots showing the average count and vertical lines ±1 standard error. The right‐hand plots show the summed maximum counts across sites, as a standard measure of overall detections in each year, constrained to those sites surveyed in all seasons.

Among the sites surveyed for *P. kundagungan* in 2016/2017 and 2020/2021, the proportion of sites occupied was 19% lower post‐fire, falling from 0.65 to 0.53 (Figure 4). Maximum counts of calling male *P. kundagungan* at occupied sites were also lower on average in 2020/2021 (mean = 11 in 2016/2017 vs. 7 in 2020/2021; Figure 4) and the summed maximum counts of calling males among the 35 sites surveyed in both seasons was substantially lower in 2020/2021 (149 vs. 247 in 2016/2017; Figure 4).

For *P. richmondensis*, the proportion of sites occupied and counts of calling males were substantially lower during surveys completed just prior to the 2019/2020 fires than in either the 2012/2013 or 2020/2021 seasons (Figure 4). The proportion of sites occupied for this species was 0.61 in 2012/2013 and 0.55 in 2020/2021, but dropped to 0.46 in the intervening season (a decline of 25% and subsequent recovery of 20%). The summed maximum counts of calling males among these sites displayed a very similar pattern, being depressed in 2019/2020 relative to 2012/2013, and although recovering in 2020/2021, remaining 14% lower than in 2012/2013 (Figure 4).

**FIGURE 5:**
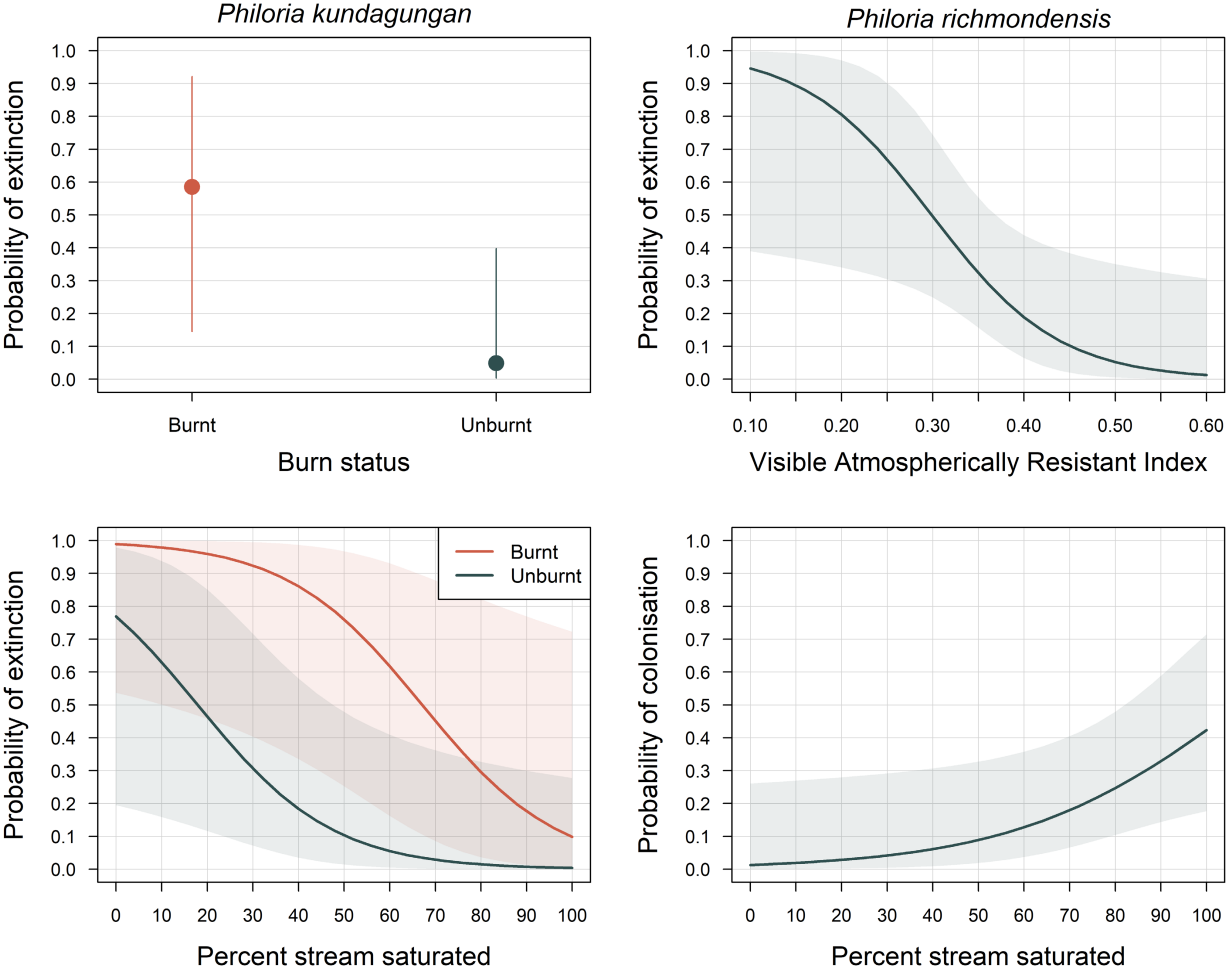
Relationships between the site‐level probability of extinction and colonization and measures of drought and fire stress for *Philoria kundagungan* and *Philoria richmondensis*. Only relationships for extinction probability are shown for *P. kundagungan*, for which the probability of colonization was modeled as a constant. Visible Atmospherically Resistant Index is a remote‐sensed spectral index that measures water availability for vegetation (hence, higher values = less drought stress). Percent stream saturated is the average value across repeat surveys at a site in 2020/2021. Shaded areas are 95% confidence intervals.

Fitting multi‐season occupancy models to these data confirmed effects of fire and drought conditions on occupancy dynamics. The top‐ranked model for *P. kundagungan* included a positive effect of a site being burnt on the probability of extinction between 2016/2017 and 2020/2021, and a negative effect of site saturation extent in 2020/2021 on this probability (Tables 5 and 6, Figure 5). For *P. richmondensis*, the top‐ranked model included a negative effect of the remote‐sensed drought severity measure Visible Atmospherically Resistant Index (VARI) on the probability of extinction (higher values = lower drought severity) and a positive effect of site saturation extent on the probability of colonization (Tables 5 and 6, Figure 5).

**TABLE 5:** The top‐ranked dynamic occupancy models for *Philoria kundagungan* and *Philoria richmondensis*. Percent stream saturated is the average value across repeat surveys at a site in 2020/2021.

Model		Log likelihood	AIC	ΔAIC	Model weight
Probability of extinction	Probability of colonization				
*P. kundagungan*					
Burn status, Percent stream saturated	Constant	−86.49	192.67	0.00	0.35
Percent stream saturated	Constant	−88.83	194.45	1.78	0.14
GEEBAM fire severity, Percent stream saturated	Constant	−87.45	194.59	1.92	0.13
Elevation, Burn status, Percent stream saturated	Constant	−86.05	194.83	2.16	0.12
Elevation, GEEBAM fire severity, percent stream saturated	Constant	−86.60	195.94	3.27	0.07
*P. richmondensis*					
Drought severity (VARI)	Percent stream saturated	−141.52	304.18	0.00	0.43
Drought severity (NDIIb6)	Percent stream saturated	−141.87	304.87	0.69	0.30
Elevation, Drought severity (VARI)	Percent stream saturated	−140.63	305.92	1.74	0.18
Elevation, Drought severity (NDIIb6)	Percent stream saturated	−141.46	307.60	3.41	0.08
Elevation	Percent stream saturated	−145.77	312.68	8.50	0.01

*Note*: Akaike's information criterion (AIC) is provided for each model, as is distance from the top model (ΔAIC). In all models, site elevation was included as a covariate for the probability of site occupancy in the initial season, with an effect of air temperature on the survey‐level probability of detection.

**TABLE 6:** Coefficient estimates for the top‐ranked dynamic occupancy model for *Philoria kundagungan* and *Philoria richmondensis*. Percent stream saturated is the average value across repeat surveys at a site in 2020/2021.

Variable	Covariate of	*P. kundagungan*		*P. richmondensis*	
		Mean	95% CI	Mean	95% CI
Elevation	Initial site occupancy probability	8.18	2.56, 13.80	2.41	0.36, 4.46
Burn status	Extinction probability	3.35	−0.11, 6.81	‐	‐
Percent stream saturated	Extinction probability	−4.14	−8.03, −0.25	‐	‐
Drought severity (VARI)	Extinction probability	‐	‐	−3.11	−5.73, −0.49
Percent stream saturated	Colonization probability	‐	‐	2.42	0.18, 4.66
Air temperature	Detection probability	−2.47	−3.56, −1.37	−1.66	−2.52, −0.79

*Note*: Mean estimates are provided along with the 95% confidence interval (95% CI).

## 4 DISCUSSION

Assessing the vulnerability of species to climate change is often completed with the aid of correlative environmental niche models that forecast gradual changes in the climatic niche space. However, the rapidity, extent and spatial pattern of declines will also be driven by amplification of the frequency and severity of climate‐related stochastic perturbations (Briscoe et al., 2016; Hoffmann, Cavanough, et al., 2021). Understanding species responses to novel stochastic events should therefore be a focus of climate change vulnerability assessments, facilitating integration of specific climate‐induced perturbations into predictive models of response (Mathewson et al., 2017; Morán‐Ordóñez et al., 2018). Our research on the effects of Australia's unprecedented “Black Summer” wildfires and the associated drought on two rainforest frogs—*P. kundagungan* and *P. richmondensis*—provides an empirical case study, and confirms for these species that deepening droughts, drying of breeding sites, and penetration of wildfires into their fire‐sensitive habitat represent important proximate threats from climate change.

Remote‐sensed mapping of fire severity suggests some 30% of potential habitat was burnt for *P. kundagungan* and 12% for *P. richmondensis*. The majority of affected areas burnt at low to moderate severity for *P. kundagungan* (71%); however, some 55% of habitat burnt for *P. richmondensis* experienced high severity fire. While persistence at burnt sites was observed for both species, and relatively high counts of calling males at burnt sites were also observed, important negative effects of fire were evident. Observed occupancy rates were substantially lower in burnt habitat for both *P. kundagungan* (14% in burnt vs. 68% in unburnt) and *P. richmondensis* (23% in burnt vs. 54% in unburnt), and there was a clear negative effect of fire on site occupancy and persistence probabilities for *P. kundagungan*, as well as counts of calling males post‐fire. These results support the recognition of unprecedented fire and changing fire regimes as a Key Threatening Process under Australia's environmental legislation (DAWE, 2022).

The lack of a clear negative effect of fire on site occupancy probability and counts for *P. richmondensis* post‐fire could be interpreted as greater resilience to fire. However, we caution that this conclusion should not be drawn from our survey data, as only a small portion of suitable habitat for this species experienced fire (12%), the majority of which was in less suitable, lower elevation areas. As such, we suggest the absence of a clear effect of fire on *P. richmondensis* is most likely the result of low statistical power to detect these effects, in turn a result of the specific nature and extent of the fire that impacted this species' habitat.

In addition to fire, impacts of drought on stream saturation extent were important correlates of the site occupancy probability and abundance of calling males post‐fire for both *P. kundagungan* and *P. richmondensis*. Likewise, we found that both remote‐sensed and field‐measured indicators of drought‐stress were important correlates of extinction and colonization probabilities in our multi‐season dataset. The multi‐season dataset is particularly useful for understanding sensitivity to moisture availability. Relative to surveys in the 2012/2013 season, occupancy rates and counts of calling males were significantly depressed for *P. richmondensis* in 2019/2020 immediately prior to the fires during a period of intense drought (Figure 4). Post‐fire, La Niña conditions produced winter and spring rainfall across much of the range of *P. richmondensis*, with a recovery in detections for this species in rehydrated streambeds. In contrast, significant rainfall deficits extended into the 2020/2021 breeding season for *P. kundagungan*, and this was clearly an important additive factor to fire in the depressed detections of this species in 2020/2021 relative to 2016/2017 (Figure 4).

Our results indicate that *Philoria* favor the coolest and wettest parts of the landscape, consistent with earlier work (Bolitho et al., 2021; Hollis, 2003; Knowles et al., 2004; Willacy, 2014). As such, forecast increases in temperature, evaporation and rainfall variability (Steffen, 2009) and reduced moisture availability from cloud‐stripping (Laidlaw et al., 2011, 2022) represent a major threat for these high‐elevation species, with no capacity for upslope migration (Bolitho & Newell, 2022). As evidenced by the 2019/2020 fires, climate drying facilitates novel fire incursion into rainforest habitats, which could cause direct frog mortality, while also degrading riparian breeding habitat. While there have been relatively few studies to assess fire related impacts on Australian frogs, Mahony et al. (2023) suggest that range restricted species that occur within fire‐sensitive vegetation communities likely incurred the highest impacts from the 2019/2020 fires. Moreover, burnt rainforests may not recover, or recovery may take many decades, due to elevated risk of repeated fire resulting from drier micro‐climate conditions associated with canopy loss (Hutley et al., 1997) and invasion of fire‐promoting weeds and or ecotonal plant species (Berry et al., 2011; Duggin & Gentle, 1998). In addition to elevated fire risk, landscape desiccation itself likely elevates recruitment failure in *Philoria*, as has been documented for other narrow‐range frog species from mesic habitats in south‐eastern (Scheele et al., 2012) and south‐western Australia (Hoffmann, Williams, et al., 2021). More broadly, climate drying and deepening droughts have been linked to declines of widespread frog species in southern Australia (Evans et al., 2020), the Rocky Mountains in the United States (McMenamin et al., 2008) and western Europe (Cayuela et al., 2016). Ultimately, in the face of unmitigated climate change, montane and/or mesic‐adapted amphibians with small geographic ranges face an uncertain future.

Nevertheless, we see several options to inform conservation planning for mesic‐adapted species at risk from climate drying and future fire events. First, ongoing monitoring provides fundamental insights into the impacts of severe drought and novel fire events, including the areas of species ranges most at risk from these events, and the ecological, demographic and genetic mechanisms through which impacts occur (see also Potvin et al., 2017; Rowley et al., 2020). Second, ecological experiments focusing on these mechanisms allows construction of models that integrate the effects of drought and fire on demographic trajectories, as well as the overarching effects of climate change on habitat extent and suitability. Penman et al. (2015) provide a useful example for an Australian forest‐adapted amphibian, coupling projections of change in habitat suitability under climate change (from a species distribution model) with a model of metapopulation viability in which an effect of increasing wildfire on survival rates was parameterized using data from planned burning operations. Third, opportunities exist to build spatial models of risk across the distribution of focal species; either mechanistic models in which severe drought and fire weather events can be simulated (e.g., Tolhurst et al., 2008), or statistical models built using remote‐sensed data on drought and fire extent (e.g., Collins et al., 2021). Taken together, research initiatives such as these offer the potential to predict the scale of the risk posed by drought and fire, and to make spatial projections of that risk. In turn, high‐risk populations and lower‐risk refugia across a species' range may be identified, facilitating spatial prioritization of conservation investments such as fire‐suppression (high‐risk populations), pre‐emptive collections for ex situ conservation (high‐risk populations) and intensive control of invasive predators or competitors (lower‐risk refugia).

Long‐term conservation planning for *P. kundagungan* and *P. richmondensis* would benefit from research initiatives such as those described above; however, there are clear actions that can be implemented now based on current knowledge. First, fire suppression is vital, ensuring recovery from recent fires, and mitigation of the risk of further fire in coming decades. Second, protection from other threatening processes must be prioritized. Logging and invasion of high‐risk weed species (such as *Lantana camara* and high biomass grasses) can significantly elevate landscape fire risk and require careful management. Likewise, feral pigs (*Sus scrofa*) are a rapidly emerging threat for *Philoria* species, with significant localized impacts observed during this study in key breeding sites (L. Bolitho, D. Newell and H. Hines, personal observation). Third, captive breeding programs should be pursued as a matter of urgency, seeking to establish founder populations that represent all topographically‐isolated populations.

Deepening droughts and novel fires represent a significant threat to *P. kundagungan* and *P. richmondensis*, just as they do to many mountain‐top and/or mesic‐adapted species susceptible to climate change (Bolitho & Newell, 2022). To conclude, we argue that both short‐ and long‐term management pathways exist to confront these threats, including ongoing monitoring to track responses to these threats, conservation planning that explicitly seeks to prepare for their impact, and targeted conservation actions that ameliorate other threatening processes and increase population resilience.

## AUTHOR CONTRIBUTIONS

**Geoffrey W. Heard:** Conceptualization (equal); data curation (lead); formal analysis (lead); investigation (equal); methodology (equal); software (lead); visualization (lead); writing – original draft (lead); writing – review and editing (equal). **Liam J. Bolitho:** Conceptualization (equal); data curation (equal); formal analysis (supporting); investigation (lead); methodology (lead); project administration (equal); visualization (supporting); writing – original draft (lead); writing – review and editing (equal). **David Newell:** Conceptualization (equal); funding acquisition (equal); methodology (lead); project administration (equal); resources (equal); supervision (equal); writing – original draft (supporting); writing – review and editing (supporting). **Harry B. Hines:** Conceptualization (equal); data curation (equal); funding acquisition (equal); investigation (equal); methodology (equal); project administration (equal); resources (equal); supervision (equal); writing – original draft (supporting); writing – review and editing (equal). **Patrick Norman:** Data curation (supporting); formal analysis (supporting); investigation (supporting); methodology (supporting); resources (supporting); software (supporting); writing – original draft (supporting); writing – review and editing (supporting). **Rosalie J. Willacy:** Investigation (supporting); methodology (supporting); writing – original draft (supporting); writing – review and editing (supporting). **Ben C. Scheele:** Conceptualization (equal); funding acquisition (equal); investigation (equal); methodology (equal); project administration (equal); supervision (equal); writing – original draft (supporting); writing – review and editing (equal).

## CONFLICT OF INTEREST STATEMENT

The authors declare no competing interests.

## Data Availability

All data and code necessary to reproduce the analysis are provided in Heard et al. (2022).
